# A potent voltage-gated calcium channel inhibitor engineered from a nanobody targeted to auxiliary Ca_V_β subunits

**DOI:** 10.7554/eLife.49253

**Published:** 2019-08-12

**Authors:** Travis J Morgenstern, Jinseo Park, Qing R Fan, Henry M Colecraft

**Affiliations:** 1 Department of PharmacologyColumbia University, Vagelos College of Physicians and SurgeonsNew YorkUnited States; 2 Department of Physiology and Cellular BiophysicsColumbia University, Vagelos College of Physicians and SurgeonsNew YorkUnited States; Weill Cornell MedicineUnited States; University of VermontUnited States

**Keywords:** calcium channel, nanobody, ubiquitin, Nedd4, calcium channel beta, cavia porcellus, Mouse

## Abstract

Inhibiting high-voltage-activated calcium channels (HVACCs; Ca_V_1/Ca_V_2) is therapeutic for myriad cardiovascular and neurological diseases. For particular applications, genetically-encoded HVACC blockers may enable channel inhibition with greater tissue-specificity and versatility than is achievable with small molecules. Here, we engineered a genetically-encoded HVACC inhibitor by first isolating an immunized llama nanobody (nb.F3) that binds auxiliary HVACC Ca_V_β subunits. Nb.F3 by itself is functionally inert, providing a convenient vehicle to target active moieties to Ca_V_β-associated channels. Nb.F3 fused to the catalytic HECT domain of Nedd4L (Ca_V_-aβlator), an E3 ubiquitin ligase, ablated currents from diverse HVACCs reconstituted in HEK293 cells, and from endogenous Ca_V_1/Ca_V_2 channels in mammalian cardiomyocytes, dorsal root ganglion neurons, and pancreatic β cells. In cardiomyocytes, Ca_V_-aβlator redistributed Ca_V_1.2 channels from dyads to Rab-7-positive late endosomes. This work introduces Ca_V_-aβlator as a potent genetically-encoded HVACC inhibitor, and describes a general approach that can be broadly adapted to generate versatile modulators for macro-molecular membrane protein complexes.

## Introduction

Inhibition of high-voltage-activated calcium channels (HVACCs) is an important prevailing or potential therapy for diverse cardiovascular (hypertension, cardiac arrhythmias, cerebral vasospasm) and neurological diseases (epilepsy, chronic pain, Parkinson’s disease) (Zamponi et al., 2015). Small molecule HVACC inhibitors include Ca_V_1 blockers (dihydropyridines, benzothiazepenes phenylalkylamines) and venom peptides that target Ca_V_2.1 (*ω*-agatoxin), Ca_V_2.2 (*ω*-conotoxin), and Ca_V_2.3 (SNX-482) channels. When introduced into an organism, small-molecule HVACC blockers are typically widely distributed leading to off-target effects that can narrow the therapeutic window and, thereby, adversely impact therapy. Genetically-encoded HVACC inhibitors can circumvent off-target effects because they can be selectively expressed in target tissues or cells; thus, they may be useful alternatives or complements to small molecule therapy (Yang et al., 2013; Murata et al., 2004).

There are seven distinct HVACCs (Ca_V_1.1 - Ca_V_1.4; Ca_V_2.1 - Ca_V_2.3) which exist in cells as multi-subunit complexes comprising pore-forming α_1_-subunits assembled with auxiliary proteins which include β, α_2_-δ, and γ subunits (Zamponi et al., 2015; Buraei and Yang, 2010; Dolphin, 2012). HVACCs are named according to the identity of the component α_1_ subunit (α_1A_-α_1F_; α_1S_) which also contains the voltage sensor, selectivity filter, and channel pore. The various auxiliary subunits typically regulate HVACC trafficking, gating, and modulation, and are recognized as potential targets for developing HVACC-directed therapeutics. For example, gabapentin, which is clinically utilized for treating epilepsy and neuropathic pain, targets HVACC α_2_-δ subunits (Gee et al., 1996). Based on the presumption that the association of α_1_ with β is obligatory for the formation of surface-targeted functional HVACCs as indicated by heterologous expression experiments (Buraei and Yang, 2010), disruption of the α_1_-β interaction has been long pursued as a strategy to develop HVACC inhibitors (Young et al., 1998; Findeisen et al., 2017; Chen, 2018; Khanna et al., 2019). To this end, over-expression of peptides derived from the α_1_-interaction domain (AID) which contains the amino acid sequence responsible for high-affinity α_1_-β association (Pragnell et al., 1994; Van Petegem et al., 2004; Chen et al., 2004; Opatowsky et al., 2003), has been utilized by several groups as putative genetically-encoded HVACC inhibitors (Findeisen et al., 2017; Yang et al., 2019). However, the efficacy of this approach in vivo may be limited as recent data suggests that in some adult tissue the α_1_-β interaction is not absolutely essential for surface trafficking of HVACCs (Yang et al., 2019; Meissner et al., 2011).

Rad/Rem/Rem2/Gem/Kir (RGK) proteins are endogenous small Ras-like G-proteins that profoundly inhibit all HVACCs when over-expressed in either heterologous cells or native tissue (Béguin et al., 2001; Finlin et al., 2003; Chen et al., 2005; Xu et al., 2010). They form ternary complexes with HVACCs via binding to constituent β subunits and inhibit currents via multiple mechanisms including removal of surface channels and impairing gating (Yang and Colecraft, 2013; Yang et al., 2010). Despite their efficacy, utility of RGKs as genetically-encoded HVACC inhibitors is confounded by potential off-target effects since they interact with and regulate other binding partners such as cytoskeletal proteins, 14-3-3, calmodulin, and CaM kinase II (Yang and Colecraft, 2013; Correll et al., 2008; Royer et al., 2018; Béguin et al., 2005; Ward et al., 2004). A critical unmet need is the development of genetically-encoded HVACC inhibitors that possess the high efficacy of RGKs but lack the problematic interactions with other signaling proteins. Here, we achieve this by fusing the homologous to the E6-AP carboxyl terminus (HECT) catalytic domain of the E3 ubiquitin ligase, neural precursor cell developmentally down-regulated protein 4 (Nedd4-2 or hereafter referred to as Nedd4L), to a Ca_V_β-targeted nanobody. The resulting construct, termed Ca_V_-aβlator, eliminated diverse HVACCs both in both reconstituted systems and native excitable cells, providing a unique new tool for probing Ca_V_1/Ca_V_2 signaling and regulation in vivo, and potential development into a therapeutic.

## Results

### Isolation and characterization of Ca_V_β-targeted nanobodies

**Figure 1.**
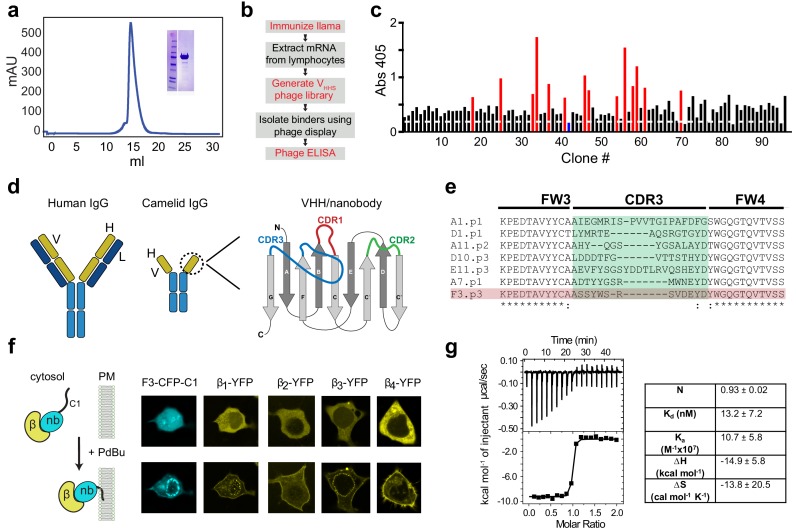
Development of a pan-Ca_V_β nanobody. (**a**) Size-exclusion chromatograph and Coomassie gel (inset) showing purified Ca_v_β_1_ from baculovirus-infected HEK293 GnTl^-^ cells. (**b**) Flow-chart of nanobody generation. (**c**) Phage ELISA using Ca_V_β_1_ as bait and periplasmic extracts from single infected *E. coli* clones. Red bars represent clones that were selected for subsequent analyses; blue bar represents a negative control from an *E. coli* expressing an anti-GFP nanobody. (**d**) Cartoon showing conventional IgG antibody (left) and camelid heavy-chain antibody (center). Right, a schematic representation of the variable heavy chain (VHH or nanobody) of camelid heavy-chain antibodies. The three CDR loops which are the primary determinants of antigen-binding are shown in red, green, and blue. (**e**) Sequence alignment of CDR3 from selected clones. (**f**) Left, schematic of co-translocation assay to determine nanobody/Ca_V_β interaction in HEK293 cells. Right, confocal images showing membrane co-translocation of Ca_V_β_X_-YFP and nb.F3-CFP-C1_PKCγ_ in response to treatment with 1 uM phorbol 12,13-dibutyrate (PdBu). (g) Left, exemplar isothermal titration calorimetry trace using purified Ca_V_β_2b_ and nb.F3. Right, summary of ITC thermodynamic parameters. N, stoichiometry; K_d_, dissociation constant; K_a_, affinity constant; ∆H, enthalpic change; ∆S entropic change. T = 298K.

We sought to develop a nanobody targeted to Ca_V_βs that would be incorporated into Ca_V_ channel complexes but be functionally silent, to serve as a vehicle to potentially address distinct enzymatic moieties or sensors to endogenous channels. We expressed Ca_V_β_1b_ and Ca_V_β_3_ in HEK293 cells using BacMam expression and purified the proteins using affinity purification, ion exchange, and size exclusion chromatography (Figure 1a). Purified β_1_ and β_3_ (1 mg each) were used for llama immunization, and successful serum conversion was confirmed by ELISA (not shown). Messenger RNA was extracted from isolated lymphocytes, PCR-amplified and cloned into a plasmid vector (pComb3XSS) to generate a V_HHS_ phage library (Figure 1b). Putative nanobody binders were enriched from the phage library using three rounds of phage display and panning (Pardon et al., 2014). We performed a 96-well ELISA on enriched phage libraries and selected 14 positive clones for sequencing (Figure 1c). We identified at least seven distinct classes of nanobody binders based on the unique sequences within complementarity determining regions (CDR1-3), the major determinants of antigen binding (Figure 1d,e).

**Figure 1—figure supplement 1.**
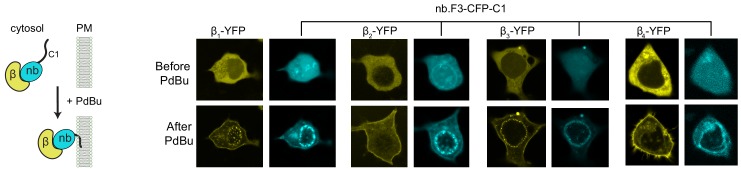
Nb.F3 binds all four Ca_V_β subunits in the cytosol of mammalian cells Left, schematic of phorbol ester 12,13-dibutyrate (PdBu) translocation assay. Right, confocal images of HEK293 cells expressing nb.F3-CFP-C1_PKCγ_ and a YFP-Ca_V_β before (top) and after (bottom) the addition of 1 µM Pdbu.

We adopted a small-molecule-induced fluorescence co-translocation assay to simultaneously determine whether: (1) individual nanobodies were well-behaved when expressed in mammalian cells (i.e. do not aggregate), and (2) bound Ca_V_βs. A tripartite construct consisting of individual nanobodies fused to CFP and the C1 domain of PKCγ was cloned into a CMV expression vector and transiently co-transfected with YFP-tagged Ca_V_βs into HEK293 cells. After pilot experiments, we chose one nanobody clone, nb.F3, for in-depth characterization and development. Both nb.F3-CFP-C1 and YFP-β_1_ were uniformly expressed in the cytosol of transfected HEK293 cells (Figure 1f). Application of 1 μM phorbol-12,13-dibutyrate (PdBu) led to the rapid and dramatic redistribution of nb.F3-CFP-C1 from the cytosol to the plasma and nuclear membranes (Figure 1f). Reassuringly, YFP-β_1_ concomitantly redistributed to the plasma and nuclear membranes, providing a convenient visual confirmation that it associates with nb.F3 inside cells (Figure 1f). Similar experiments conducted with the other Ca_V_βs (β_2_-β_4_) showed that they all bind with nb.F3-CFP-C1 in cells (Figure 1f; Figure 1—figure supplement 1), indicating the nanobody interacts with an epitope conserved among Ca_V_βs. Isothermal titration calorimetry using purified nb.F3 and Ca_V_β_2b_ indicated a high-affinity (*K*_d_ = 13.2 ± 7.2 nM) interaction and a 1:1 stoichiometry (Figure 1g).

**Figure 2.**
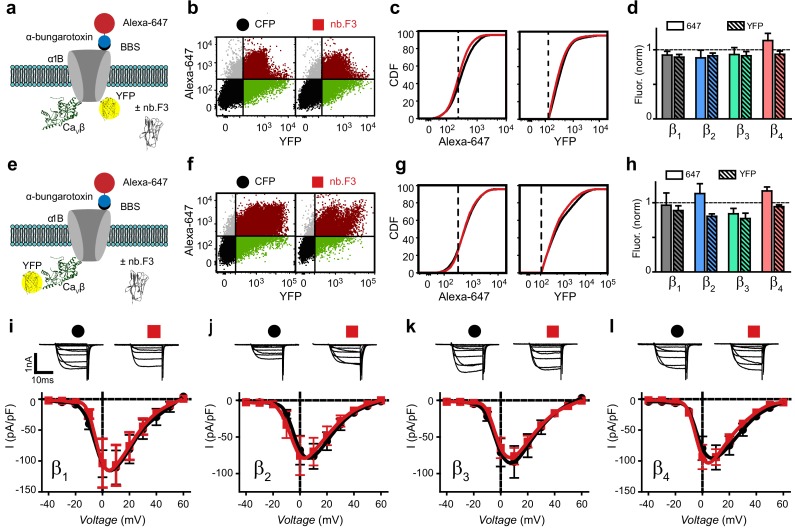
Nb.F3 is functionally silent on reconstituted Ca_V_2.2 channels. (**a**) Schematic of experimental strategy; BBS-α_1B_-YFP was transfected in HEK293 cells with α_2_δ, Ca_V_β and either CFP or nb.F3-P2A-CFP. (**b**) Exemplar flow cytometry dot plot of cells expressing BBS-α_1B_-YFP + Ca_V_β_1_ + α_2_δ-1 and either CFP (left) or nb.F3-P2A-CFP (right). Approximately 100,000 cells are represented here and throughout. Horizontal and vertical lines represent the threshold for YFP- and Alexa-647-positive cells, respectively, as determined with single color controls. (**c**) Cumulative distribution histogram of Alexa-647 (left) or YFP fluorescence (right) from CFP (black) or nb.F3 (red) expressing cells. YFP-positive cells were selected for the analysis; dashed lines represent thresholds for Alexa-647 and YFP fluorescence signals above background. (**d**) Summary flow cytometry data of surface (647, filled) and total (YFP, patterned) levels of BBS-α_1B_-YFP. Data from nb.F3-expressing cells was normalized to CFP control group. n=>5,000 cells analyzed per experiment, N=4 separate experiments, error bars, s.e.m. (**e**) Experimental strategy; HEK293 cells were transfected with BBS-α_1B_ + Ca_V_β-YFP + α_2_δ-1. (**f-h**) Same format as (**b-d**) for cells expressing BBS-α_1B_ + Ca_V_β-YFP+ α_2_δ-1 ± nb.F3-P2A-CFP. (i) Exemplar whole-cell Ba^2+^ currents (top) and population *I-V* curves (bottom) in HEK293 cells expressing α_1B_ + Ca_V_β_1_ + α_2_δ-1 and either CFP (black) or nb.F3-P2A-CFP (red). (j-l) Same format as (i) for cells expressing Ca_V_β_2_ (j), Ca_V_β_3_ (k), and Ca_V_β_4_ (l). Scale bar 1 nA, 10 ms. Data are means ± s.e.m., n=10 for each point.

It was important to our overall strategy that nb.F3 incorporate into assembled HVACC complexes without impacting channel function or subunit stability. We utilized a flow cytometry assay to assess the impact of nb.F3 on recombinant Ca_V_2.2 trafficking, subunit expression levels, and whole-cell currents, all of which are known to be regulated by Ca_V_β (Figure 2) (Waithe et al., 2011). We used an engineered α_1B_ harboring two tandem high-affinity bungarotoxin-binding sites (2XBBS) in the extracellular domain IV S5-S6 loop and a C-terminus YFP tag to enable simultaneous detection of surface (Alexa647-conjugated bungarotoxin) and total (YFP fluorescence) channel populations in non-permeabilized cells (Figure 2a). We co-expressed BBS-α_1B_-YFP and Ca_V_β either with or without nb.F3-P2A-CFP and utilized flow cytometry to rapidly measure surface and total channel expression. In cells expressing BBS-α_1B_-YFP and β_1b_, nb.F3 had no impact on Alexa647 or YFP fluorescence compared to control (Figure 2b–d), indicating no disruption of channel trafficking or effect on α_1B_ expression. Similar results regarding the inertness of nb.F3 on α_1B_ trafficking and stability were obtained when Ca_V_2.2 was reconstituted with the other Ca_V_β (β_2_-β_4_) subunits (Figure 2d).

**Figure 2—figure supplement 1.**
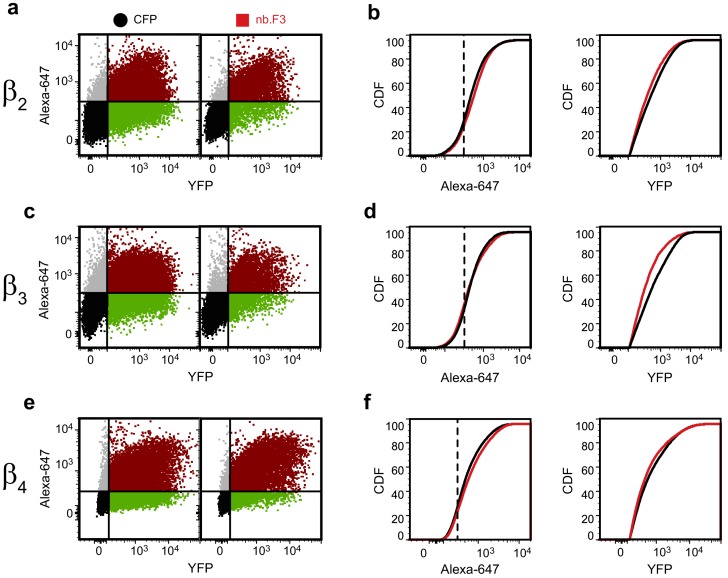
Exemplar flow cytometry data for BBS-α_1B_ with YFP-Ca_V_β_2_- Ca_V_β_4._ (**a**) Exemplar flow cytometry dot plot of cells transfected with BBS-α_1B_, α_2_-δ, YFP-Ca_V_β_2_ and CFP (left) or nb.F3 (right). (**b**) Cumulative distribution histogram of data from (**a**) of Alexa-647 (left) or YFP fluorescence (right) from CFP (black) or nb.F3 (red) expressing cells. YFP-positive cells (n => 5,000 cells/experiment) were selected for analysis; the threshold for 647 labeling and YFP fluorescence above background is represented with the dashed line. (**c,d**) as in (**a,b**) for cells expressing YFP-Ca_V_β_3_. (**e,f**) as in (**a,b**) for cells expressing Ca_V_β_4._.

**Figure 2—figure supplement 2.**
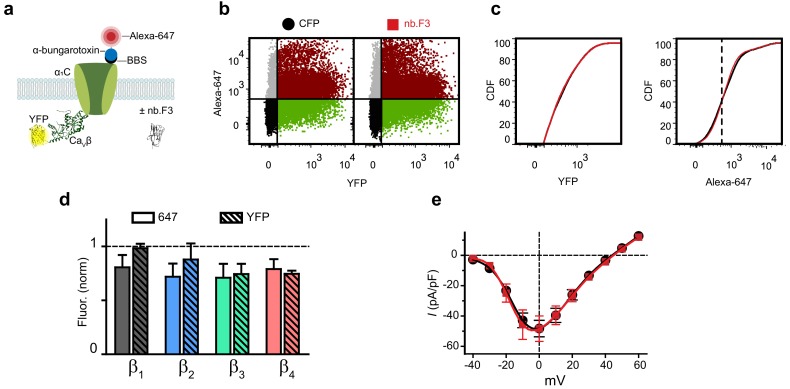
Nb.F3 is functionally silent on reconstituted Ca_V_1.2 channels. (**a**) Cartoon of experimental strategy. BBS-α_1C_ was transfected in HEK293 cells with each YFP-Ca_V_β and either CFP or nb.F3-P2A-CFP. (**b**) Exemplar flow cytometry dot plot of cells transfected with BBS-α_1C_, Ca_V_β_1_ and CFP (left) or nb.F3 (right). (**c**) Cumulative distribution histogram of Alexa-647 (left) or YFP fluorescence (right) from CFP (black) or nb.F3 (red) expressing cells. YFP-positive cells (n > 5,000 cells/experiment) were selected for analysis; the threshold for 647 labeling and YFP fluorescence above background is represented with the dashed line. (**d**) Summary flow cytometry data of surface (647, filled) and total (YFP, patterned) levels of BBS-α_1C_. Data from nb.F3 was normalized to CFP control group. N = 4 separate experiments, error bars, s.e.m. (**e**) population I-V curves from whole-cell patch clamp measurements in HEK293 cells expressing α_1C_, Ca_V_β_2a_, and CFP (black, n = 9) or nb.F3 (red, n = 12). Data are means ± s.e.m.

To examine a potential direct impact of nb.F3 on Ca_V_β itself, we applied the flow cytometry assay to cells expressing BBS-α_1B_ + β-YFP ± nb .F3-P2A-CFP (Figure 2e). Not surprisingly, nb.F3 did not impact the surface trafficking of BBS-α_1B_ co-expressed with any of the four Ca_V_β isoforms (Figure 2f–h, Figure 2—figure supplement 1). The expression levels of β_1_-YFP and β_4_-YFP were unaffected by nb.F3, whereas the levels of β_2_ and β_3_ were modestly reduced (although this effect did not reach statistical significance), suggesting a possible slightly increased vulnerability of these two isoforms to degradation when bound by the nanobody (Figure 2h). Similar observations regarding the lack of effect of nb.F3 on channel trafficking and subunit expression levels were made in cells expressing Ca_V_1.2 channels reconstituted from BBS-α_1C_ + β-YFP ± nb .F3-P2A-CFP (Figure 2—figure supplement 2).

Finally, we used patch-clamp electrophysiology to evaluate the impact of nb.F3 on whole-cell currents through recombinant Ca_V_2.2 channels reconstituted in HEK293 cells. Cells expressing α_1B_ + β_1b_ + α_2_δ displayed robust whole-cell Ba^2+^ currents that were completely unaffected by nb.F3 (Figure 2i; *I_peak,0mV_* = −104.4 ± 22.0 pA/pF, n = 10 for CFP, and *I_peak,0mV_* = −103.5 ± 39.5 pA/pF, n = 10 for nb.F3). A similar lack of effect of nb.F3 was observed on currents from either Ca_V_2.2 reconstituted with the other β_2_-β_4_ subunits (Figure 2j-l), or Ca_V_1.2 (α_1C_ + β_2a_ + α_2_δ) channels (Figure 2—figure supplement 2).

Overall, these results indicate that nb.F3 binds β_1_-β_4_ subunits in cells, and is potentially assembled into Ca_V_ channel complexes in a functionally silent manner, essentially acting as an unobtrusive passenger. However, it was also possible that the apparent functional inertness of nb.F3 on Ca_V_2.2 and Ca_V_1.2 channels had a more trivial explanation— that Ca_V_βs assembled with pore-forming α_1_-subunits are simply inaccessible to nb.F3. We could discriminate between these two possible scenarios by determining whether nb.F3 could be used to target bioactive molecules to regulate assembled channels, as we did next.

### Potent functional effects of an F3-Nedd4L chimeric protein on Ca_V_1/Ca_V_2 channels

We hypothesized that fusing the catalytic domain of an E3 ubiquitin ligase to nb.F3 would generate a genetically-encoded molecule that inhibits Cav1/Cav2 channels by reducing their surface density (Kanner et al., 2017). Accordingly, we generated a chimeric construct (nb.F3-Nedd4L) by fusing the catalytic HECT domain of Nedd4L to the C-terminus of nb.F3. We also generated a catalytically dead mutant of the chimeric construct (nb.F3-Nedd4L[C942S]) to distinguish between ubiquitination-dependent and independent effects. Both constructs were generated in a P2A-CFP expression vector, enabling use of CFP fluorescence to confirm protein expression.

**Figure 3.**
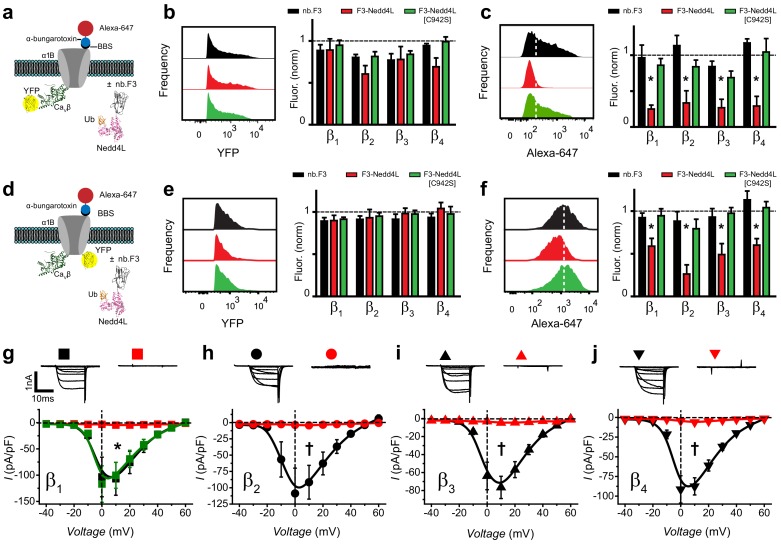
Functional impact of a chimeric nb.F3-Nedd4L protein (Ca_V_-aβlator) on reconstituted Ca_v_2.2 channels. (**a**) Schematic of experimental design; HEK293 cells were transfected with BBS-α_1B_ + Ca_V_β-YFP + α_2_δ−1, and either nb.F3, nb.F3-Nedd4L, or nb.F3-Nedd4L[C942S]. (**b**) Exemplar histograms (left) and summary data (right) of flow cytometry experiments measuring total (YFP) levels of Ca_V_β_1b_-YFP. Each data set was normalized to a control group that expressed CFP. n > 5,000 cells analyzed per experiment, N = 3 separate experiments, error bars, s.e.m. (**c**) Exemplar histograms (left) and summary data (right) of flow cytometry experiments measuring surface (647) levels of BBS-α_1B_. White dashed line is the threshold for 647 signal above background. (**d**) Experimental strategy; same format as in (**a**) except YFP was fused to BBS-α_1B_, enabling measurement of the total levels of the α_1B_ subunit. (**e-f**) Same format as in (**b-d**) for cells expressing BBS-α_1B_-YFP + Ca_V_β + α_2_δ-1. (**g**) Exemplar traces (top) and population *I-V* curves (bottom) from whole-cell patch clamp measurements in HEK293 cells expressing α_1B_ + Ca_V_β_1b_ + α_2_δ-1 and nb.F3 (black, *I_peak, 0mV_* = -103.5 ± 39.5 pA/pF, n=10), nb.F3-Nedd4L (red, *I_peak, 0mV_* = -3 ± 0.53 pA/pF, n=11), or nb.F3-Nedd4L[C942S] (green, *I_peak, 0mV_* = -117 ± 34.8 pA/pF, n=8). (**h-j**) Same format as (**g**) for Ca_V_2.2 channels reconstituted with Ca_V_β_2_ (**h**), Ca_V_β_3_ (**i**), and Ca_V_β_4_ (**j**) with nb.F3 (black) or nb.F3-Nedd4L (red). Scale bar 1nA, 10ms. Data are means ± s.e.m., n=10 for each point. *P < 0.05 compared with control, one-way ANOVA with Tukey’s multiple comparison test. †P < 0.01 compared with control, unpaired, two-tailed Student’s t-test.

In experiments mimicking those described for nb.F3, we examined the impact of nb.F3-Nedd4L and nb.F3-Nedd4L[C942S] on reconstituted Ca_V_2.2 channel trafficking, subunit expression levels, and whole-cell currents (Figure 3). Given the classical role of E3 ubiquitin ligases in mediating degradation of target proteins, we first assessed if nb.F3-Nedd4L affected total Ca_V_β expression (Figure 3a,b). In cells expressing BBS-α_1B_ + β_1b_-YFP + α_2_δ, neither F3-Nedd4L nor F3-Nedd4L[C942S] had any significant impact on β_1b_ total expression as reported by the unchanged YFP fluorescence compared to negative control cells (Figure 3a,b). Similar results were obtained when BBS-α_1B_ was reconstituted with YFP-tagged β_2_, β_3_, or β_4_ subunits, though there was a trend towards lower fluorescence with β_2a_ and β_4_ (Figure 3b). By contrast, nb.F3-Nedd4L significantly suppressed surface density of BBS-α_1B_ irrespective of the identity of the co-expressed YFP-tagged Ca_V_β (Figure 3c, red bars; Figure 3—figure supplement 1). The decreased BBS-α_1B_ surface density was not observed with nb.F3-Nedd4L[C942S] (Figure 3c, green bars), indicating it requires the catalytic activity of the attached Nedd4L HECT domain. Similarly, in cells expressing BBS-α_1C_ + β_X_-YFP, nb.F3-Nedd4L strongly reduced Ca_V_1.2 surface density in a ubiquitin-dependent manner (Figure 3—figure supplement 2).

**Figure 3—figure supplement 1.**
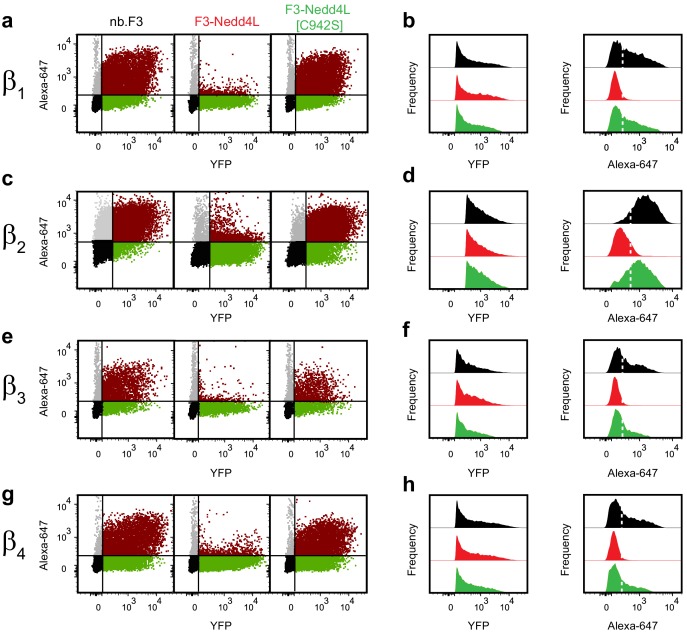
Exemplar flow cytometry data for Ca_V_-aβlation of BBS-α_1B_ with YFP-Ca_V_β_2_- Ca_V_β_4._ (**a**) Exemplar flow cytometry dot plot of cells transfected with BBS-α_1B_, α_2_-δ, YFP-Ca_V_β_1_ and nb.F3 (left), nb.F3-Nedd4L (middle) or nb.F3-Nedd4L[C942S] (right). (**b**) Histogram a of YFP (left) or Alexa-647 fluorescence (right) from samples in (**a**) YFP-positive cells (n > 5,000/experiment) were selected for analysis, the threshold for 647 labeling above background is represented with the dashed line. (**c,d**) as in (**a,b**) for cells expressing YFP-Ca_V_β_2_. (**e,f**) as in (**a,b**) for cells expressing YFP-Ca_V_β_3_. (c,d) as in (a,b) for cells expressing YFP-Ca_V_β_2_. (**g,h**) as in (a,b) for cells expressing YFP-Ca_V_β_4_.

**Figure 3—figure supplement 2.**
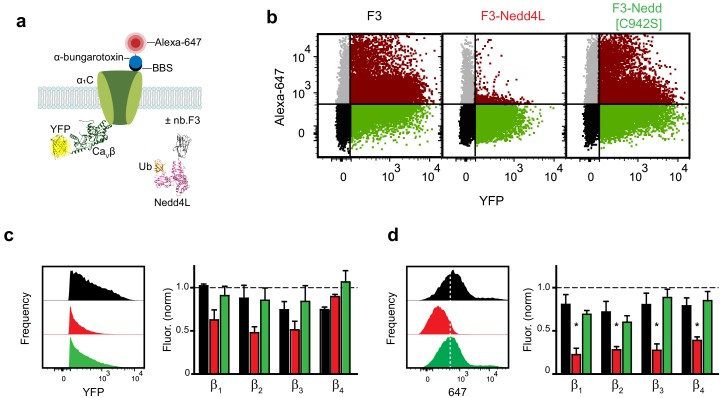
Functional impact of a chimeric nb.F3-Nedd4L protein (Ca_V_-aβlator) on reconstituted Ca_v_1.2 channels. (**a**) Schematic of experimental design. HEK293 cells were transfected with BBS-α_1C_, YFP-Ca_V_β, and either nb.F3, nb.F3-Nedd4L or nb.F3-Nedd4L[C942S]. (**b**) Exemplar flow cytometry dot plot of cells transfected with BBS-α_1C_, YFP-Ca_V_β_1b_ and nb.F3 (left), nb.F3-Nedd4L (middle) or nb.F3-Nedd4L[C942S] (right). (**c,d**) Histogram of YFP (**c**) and Alexa-647 (**d**) fluorescence from samples in (**b**) (left) and summary data from N = 3 separate experiments (right). YFP-positive cells (n > 5,000 cells per experiment) were selected for analysis, the threshold for 647 labeling above background is represented with the dashed line. *p<0.05 compared with control, one-way ANOVA with Tukey’s multiple comparison test.

Given the striking effect of nb.F3-Nedd4L on surface population of channels without affecting total levels Cavβ, we next assessed whether there was any impact of nb.F3-Nedd4L on total α_1B_ subunit expression. Similar to our observations for Ca_V_β, nb.F3-NeddL had no significant impact on the expression of BBS-α_1B_-YFP (Figure 3e, red bars) relative to either negative controls (black bars) or cells expressing nb.F3-Nedd4L[C942S] (green bars). Not surprisingly, nb.F3-Nedd4L markedly impaired surface trafficking of BBS-α_1B_-YFP co-expressed with any Ca_V_β (Figure 3f).

**Figure 4.**
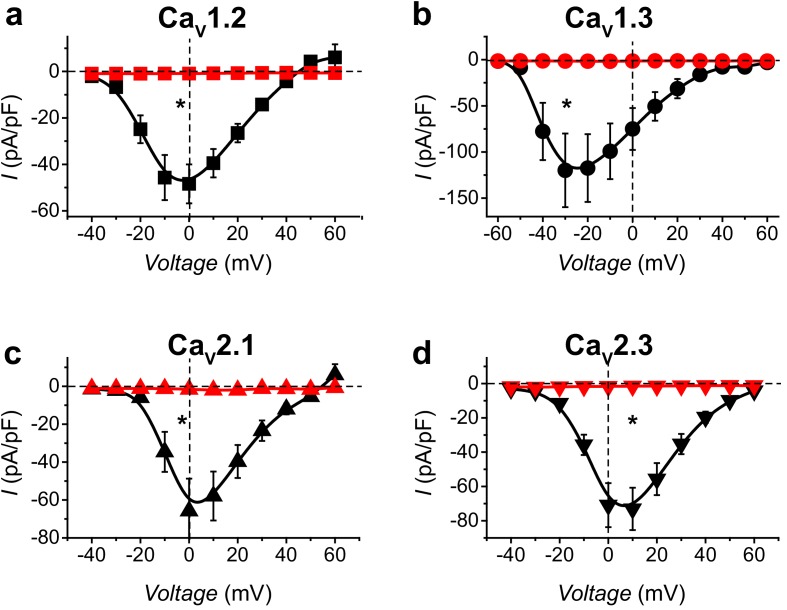
Ca_V_-aβlator inhibits distinct reconstituted HVACCs. (**a**) Population I-V curves from HEK293 expressing α_1C_ + β_1b_ + α_2_δ−1 with either nb.F3 (black, *I_peak, 0mV_* = −48.4 ± 8.4 pA/pF, n = 12) or Ca_V_-aβlator (red, *I_peak, 0mV_* = −0.93 ± 0.16 pA/pF, n = 8). (**b-d**) Same format as (**a**) for cells expressing reconstituted Ca_V_1.3 (**b**), Ca_V_2.1 (**c**), or Ca_V_2.3 (**d**) channels. Data are means ± s.e.m.†p<0.01 compared with control, unpaired, two-tailed Student’s t-test.

Finally, we examined the functional impact of nb.F3-Nedd4L on reconstituted Ca_V_2.2 whole-cell currents. Remarkably, nb.F3-Nedd4L essentially eliminated Ca_V_2.2 currents reconstituted from α_1B_ + α_2_δ co-expressed with any of the four Ca_V_βs (Figure 3g-j). Further, nb.F3-Nedd4L was equally effective in ablating whole-cell currents in reconstituted Ca_V_1.2, Ca_V_1.3, Ca_V_2.1, and Ca_V_2.3 channels (Figure 4).

Given its exceptional efficacy in ablating whole-cell HVACC currents via a functionalized Ca_V_β-targeted nanobody, we named nb.F3-Nedd4L as Ca_V_-aβlator, and describe the process of HVACC current elimination by this molecule as Ca_V_-aβlation.

### Ca_V_-aβlation of endogenous Ca_V_1.2 channels in cardiomyocytes

**Figure 5.**
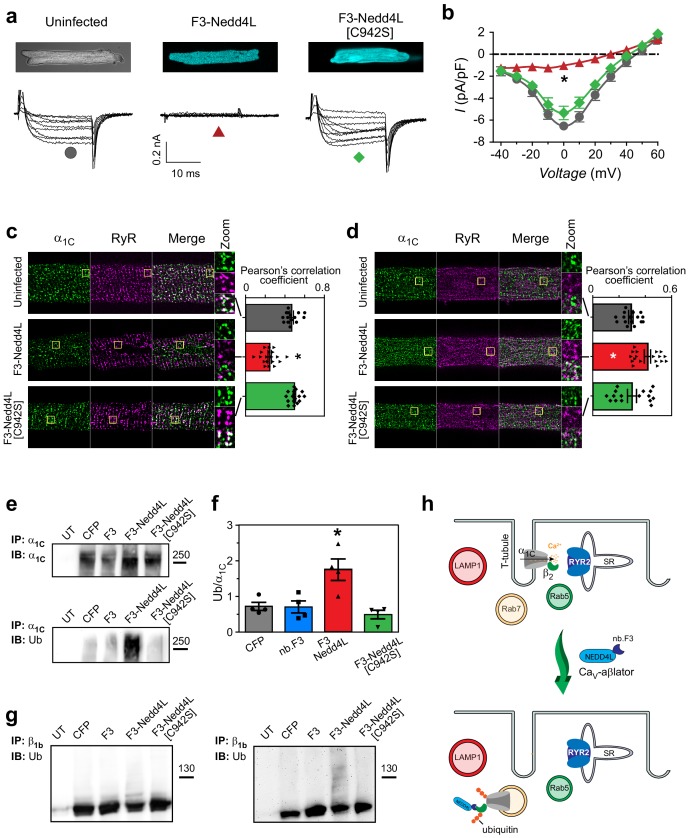
Ca_V_-aβlation of endogenous Ca_V_1.2 in cardiomyocytes. (**a**) Confocal images (top) and exemplar traces from whole-cell recordings of uninfected guinea pig cardiomyocytes (left), or infected with adenovirus expressing either Ca_V_-βlator (middle) or nb.F3-Nedd4L[C942S] (right). Scale bar 0.2nA, 10 ms. (**b**) Population I-V curves from cardiomyocytes expressing Ca_V_-βlator (red), nb.F3-Nedd4L[C942S] (green), or an uninfected control (black). (**c**) Left, exemplar confocal images of cardiomyocytes fixed and immunostained with α_1C_ (green) and ryanodine receptor (RyR2, magenta) antibodies. Yellow box indicates region of high-zoom merge image.Right, co-localization between α_1C_ and RyR in uninfected cardiomyocytes (gray, PCC = 0.47 ± 0.02, n = 15), and those expressing either Ca_V_-aβlator (red, PCC = 0.24 ± 0.02 n = 19), or nb.F3-Nedd4L[C942S] (green, PCC = 0.50 ± 0.01, n = 17). (**d**) Left, exemplar confocal images of fixed cardiomyocytes immunostained with α_1C_ (green) and Rab7 (magenta) antibodies. Yellow box indicates region of high-zoom merge image. Right, colocalization between α_1C_ and Rab7 in uninfected cardiomyocytes (gray, PCC = 0.29 ± 0.02, n = 16), and those expressing either Ca_V_-aβlator (red, PCC = 0.42 ± 0.02, n = 18), or nb.F3-Nedd4L[C942S] (green, PCC = 0.30 ± 0.03, n = 16). (**e**) Pulldown of α_1C_ in HEK293 cells expressing α_1C_, β_1b_ and either CFP, nb.F3, Ca_V_-aβlator, or nb.F3-Nedd4L-[C942S]. Top, probing pulldown with α_1C_ antibody. Bottom, same blot stripped and re-probed with ubiquitin antibody. (**f**) Quantification of four separate experiments, as performed in (**e**). Data are means ± s.e.m for each point. *p<0.05 compared to control, one-way ANOVA with Tukey’s multiple comparison test. (**g**) Pulldown of Ca_V_β_1b_, as in (**e**). Left, probing with Ca_V_β_1b_. Right, same blot stripped and re-probed with ubiquitin antibody. (**h**) Cartoon illustrating Ca_V_-aβlator-induced relocation of Ca_V_1.2 from dyads to Rab7-positive late endosomes in cardiomyocytes.

We next determined whether Ca_V_-aβlator could effectively inhibit HVACC currents in native cells where the nano-environment around Ca_V_1/Ca_V_2 channels is typically more complex than in heterologous cells. Cultured adult guinea pig ventricular cardiomyocytes (CAGPVCs) provided an initial exceptional challenge because they have an intricate cyto-architecture and express Ca_V_1.2 channels that are predominantly targeted to specialized dyadic junctions. Moreover, as it has now been shown that in adult cardiomyocytes binding of α_1C_ to Ca_V_β is not obligatory for substantive Ca_V_1.2 channel trafficking to the surface sarcolemma (Yang et al., 2019; Meissner et al., 2011), the fraction of Ca_V_β-bound Ca_V_1.2 channels contributing to the whole-cell L-type current (*I*_Ca,L_) in ventricular myocytes is ambiguous. We used adenovirus to express Ca_V_-aβlator or nb.F3-Nedd4L[C942S] in CAGPVCs which retain the rod-shaped phenotype and overall cyto-architecture of freshly isolated heart cells (Figure 5a). Control (non-infected) cardiomyocytes expressed *I*_Ca,L_ that peaked at a 0 mV test pulse (Figure 5a,b; *I_peak,0mV_* = −6.5 ± 0.2 pA/pF, n = 8). By contrast, in contemporaneous experiments, cardiomyocytes expressing Ca_V_-aβlator via adenovirus-mediated infection displayed virtually no Ca_V_1.2 currents, demonstrating an exceptional Ca_V_-aβlation efficiency in this system (Figure 5a,b; *I_peak,0mV_* = −1.0 ± 0.3 pA/pF, n = 9). Cardiomyocytes expressing nb.F3-Nedd4L[C942S] displayed *I*_Ca,L_ similar to control (*I_peak,0mV_* = −5.1 ± 0.6 pA/pF, n = 10), indicating that ubiquitination is necessary for Ca_V_-aβlation in this system.

**Figure 5—figure supplement 1.**
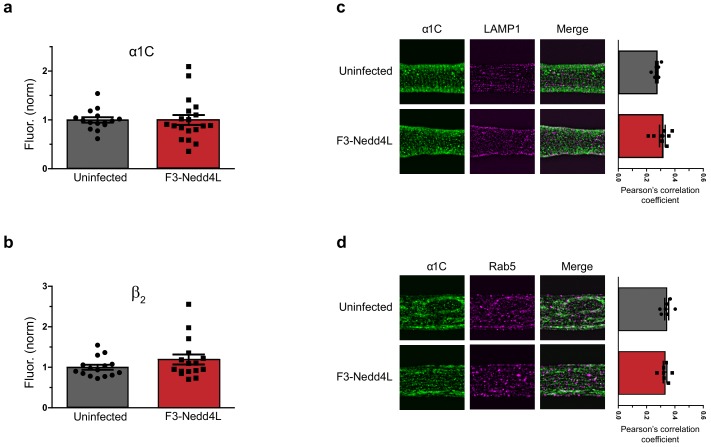
Ca_V_-aβlator does not redistribute α_1C_ to Rab5 early-endosomes or lysosomes, nor decrease total levels of α_1C_ or β_2_. (**a**) Comparison of total α_1C_ levels in uninfected cardiomyocytes (gray, n = 15) and those infected with nb.F3-Nedd4L (red, n = 19). (**b**). Comparison of total β_2_ levels in uninfected cardiomyocytes (gray, n = 15) and those infected with nb.F3-Nedd4L (red, n = 16), normalized to uninfected controls. Data are means ± s.e.m. (**c**) Left, exemplar confocal images of uninfected (top) or F3-Nedd4L-infected (bottom) guinea pig cardiomyocytes, fixed and immunostained with antibodies towards α_1C_ (left) and LAMP1 (middle). Right, colocalization between α_1C_ and Rab5 in uninfected cardiomyocytes (gray, n = 7) and those expressing F3-Nedd4L (red, n = 7). Data are means ± s.e.m. (**d**) as in (**c**), showing immunostaining and colocalization analysis of cardiomyocytes immunostained against α_1C_ and Rab5.

What is the mechanism of Ca_V_-aβlation in cardiomyocytes? We used immunofluorescence to probe how Ca_V_-aβlator affected expression levels and sub-cellular localization of Ca_V_1.2 α_1C_ and β_2_ subunits, respectively, in cardiomyocytes. Ca_V_α_1C_ in uninfected cardiomyocytes presented with a characteristic striated punctate distribution pattern that co-localized with that of ryanodine (RyR2) receptors (Figure 5c), reflecting their well-known predominant localization at dyadic junctions (Scriven et al., 2000; Bers, 2002). A similar distribution pattern for α_1C_ was observed in cardiomyocytes expressing nb.F3-Nedd4L[C942S], consistent with the lack of effect of this protein on *I*_Ca,L_. In cardiomyocytes expressing Ca_V_-aβlator, the signal intensity for punctate α_1C_ staining was unchanged from control cells (Figure 5—figure supplement 1), suggesting no impact of the presumed increase in ubiquitination on the stability of the protein. However, there was a redistribution of α_1C_ from dyadic junctions, as reported by a dramatic loss of co-localization between α_1C_ and RyR2 (Figure 5c). Rather, the punctate α_1C_ signals in Ca_V_-aβlator-expressing cardiomyocytes coincided with Rab7, but not Rab5 or LAMP1, immunofluorescence signals (Figure 5d; Figure 5—figure supplement 1). Thus, the mechanism of Ca_V_-aβlator inhibition of *I*_Ca,L_ is redistribution of α_1C_ from dyadic junctions to intracellular compartments, specifically Rab7-positive late endosomes (Figure 5h) (Rink et al., 2005).

Cardiomyocytes expressing Ca_V_-aβlator also showed no difference in total Ca_V_β_2_ levels as compared to either uninfected or nb.F3-Nedd4L[C942S]-expressing cells (Figure 5—figure supplement 1). Hence, Ca_V_-aβlator-mediated redistribution of Ca_V_1.2 in cardiomyocytes cannot be explained as simply due to an absence of Ca_V_β. An intriguing possibility was that though Ca_V_-aβlator is specifically targeted to Ca_V_β in channel complexes, it is also able to directly catalyze ubiquitination of α_1_ subunits within the macro-molecular complex. Indeed, in pulldown experiments of recombinant Ca_V_1.2 channels, Ca_V_-aβlator substantially increased ubiquitination of both α_1C_ (Figure 5e,f) and Ca_V_β_1b_ subunits (Figure 5g). Nevertheless, the overall levels of α_1C_ expression was unchanged with Ca_V_-aβlator despite the increased ubiquitination (Figure 5e). Taken together, our results suggest that direct ubiquitination of α_1C_ by Ca_V_-aβlator may underlie the redistribution of Ca_V_1.2 channels from dyads to Rab7-positive late endosomes (Figure 5h).

### Ca_V_-aβlation in dorsal root ganglion (DRG) neurons and pancreatic β cells

**Figure 6.**
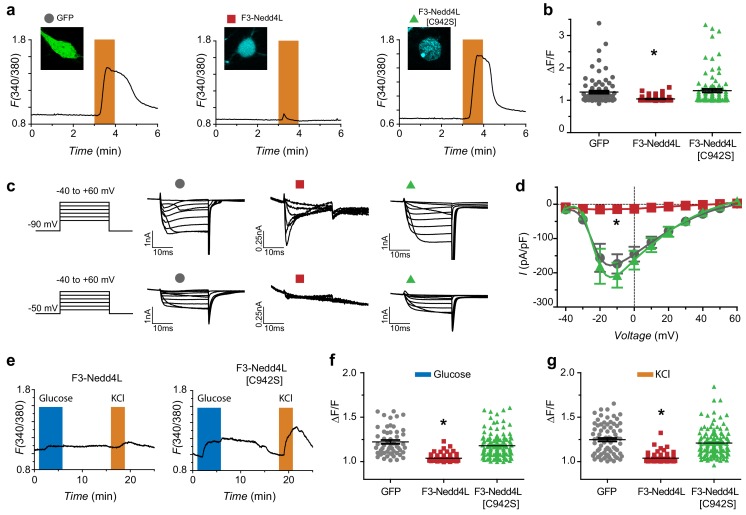
Ca_V_-aβlation of HVACCs in DRG neurons and pancreatic β-cells. (**a**) Exemplar Fura-2 traces of murine DRG neurons infected with GFP (left), F3-Nedd4L (middle), F3-Nedd4L[C942S] (right), with confocal images in inset. The orange bars represent depolarization with 40 mM KCl. (**b**) Summary of maximum responses from neurons infected with GFP (Peak response = 1.25 ± 0.04, n = 84), F3-Nedd4L (1.04 ± 0.01, n = 77), and F3-Nedd4L[C942S] (1.30 ± 0.05, n = 92) in response to 40 mM KCl. Peaks were normalized to the baseline, defined as 1 min prior to the addition of KCl. (**c**) Exemplar traces of DRG neurons infected with GFP (left), F3-Nedd4L (middle), F3-Nedd4L[C942S] (right). Traces were collected at both a holding potential of −90 mV (top) and −50 mV (bottom). Notably, Ca_V_-aβlator-infected neurons still show robust T-type current when held at −90 mV. (**d**) Population I-V curves from DRG neurons infected as in (**a**). Measurements were made at a holding potential of −90 mV. Symbols are mean currents calculated from 15 to 20 ms of a 20 ms test pulse. Data are means ± s.e.m. (**e**) Exemplar fura-2 traces from dispersed pancreatic islets infected with Ca_V_-aβlator (left) or F3-Nedd4L[C942S] (right) challenged with 16.8 mM glucose (blue bars) and 40 mM KCl (orange bars). (**f**) Summary of maximum responses from pancreatic β-cells infected with GFP (Peak response = 1.22 ± 0.02, n = 53), F3-Nedd4L (Peak response = 1.04 ± 0.01, n = 62), and F3-Nedd4L[C942S] (Peak response = 1.18 ± 0.01, n = 122) in response to 16.8 mM glucose. (**g**) Summary of maximum responses from pancreatic β-cells infected with GFP (Peak response = 1.25 ± 0.02, n = 77), F3-Nedd4L (Peak response = 1.04 ± 0.01, n = 62), and F3-Nedd4L[C942S] (Peak response = 1.21 ± 0.01, n = 122) in response to 40 mM KCl. Data are means ± s.e.m. *p<0.05 compared to control, one-way ANOVA with Tukey’s multiple comparison test.

We next tested the efficacy of Ca_V_-aβlator to suppress HVACCs in murine dorsal root ganglion (DRG) neurons which were of interest because they express multiple Ca_V_1/Ca_V_2 channel types (Murali et al., 2015; McCallum et al., 2011), and also play a key role in the processing of noxious signals including pain and itch (Han et al., 2013; Kim et al., 2016). We infected cultured DRG neurons with adenovirus expressing either GFP, Ca_V_-aβlator, or nb.F3-Nedd4L[C942S]. Given their heterogeneous nature, we first used fura-2 to measure calcium influx into a population of DRG neurons in response to depolarization with 40 mM KCl (Figure 6a,b). Recordings were done in the presence of 5 μM mibefradil to block low-voltage-activated T-type calcium channels which are also prevalent in these cells (Puckerin et al., 2018; Jagodic et al., 2008). In neurons expressing GFP or nb.F3-Nedd4L[C942S], a substantial fraction of cells displayed large increases in fura-2-reported Ca^2+^ transients in response to 40 mM KCl, indicating the opening of Ca_V_1/Ca_V_2 channels (Figure 6a,b). By contrast, depolarization-induced Ca^2+^ influx was virtually eliminated in neurons expressing Ca_V_-ablator, demonstrating highly efficient Ca_V_-ablation in this system (Figure 6a,b).

We used whole-cell patch clamp to further characterize the impact of Ca_V_-aβlator on calcium currents in DRG neurons. It was of particular interest to determine relative effects of Ca_V_-aβlator on HVACCs and LVA T-type channels that are present in a subset of DRG neurons. We recorded families of whole-cell currents evoked by test pulses (from −40 mV to +60 mV in 10 mV increments) from a holding potential of either −90 mV or −50 mV to inactivate any T-type channel present (Figure 6c). Cells expressing GFP (control) or F3-Nedd4L[C942S] displayed large *I*_Ba_ irrespective of the holding potential (Figure 6c,d; *I_peak,-10mV_* = −173.9 ± 28.2 pA/pF, n = 6 for GFP, *I_peak,-10mv_* = −206.7 ± 36.4 pA/pF, n = 5 for F3-Nedd4L[C942S]), though those recorded with a −50 mV holding potential had a lower amplitude reflecting inactivation of T-type channels and also a fraction of HVACCs. Cells expressing Ca_V_-aβlator displayed essentially no HVACC currents (Figure 6c,d; *I_peak,-10mV_* = −14.3 ± 6.2 pA/pF), most evident as an absence of *I*_Ba_ recorded from a −50 mV holding potential (Figure 6c, middle). Moreover, in these cells, when currents were recorded from a −90 mV holding potential, they displayed fast inactivation kinetics characteristic of T-type channels (Figure 6c). Overall, these results indicate Ca_V_-ablator selectively eliminates HVACCs in DRG neurons without impacting LVA T-type channels.

Finally, we tested whether Ca_V_-ablator is also effective in murine pancreatic β-cells, which have multiple Ca_V_ channel types (Ca_V_1.2, Ca_V_1.3, and Ca_V_2.1) involved in insulin release (Yang and Berggren, 2006). We used adenovirus to infect digested islets isolated from transgenic mice expressing tdTomato in pancreatic β-cells. Control cells expressing GFP or nb.F3-Nedd4L[C942S] displayed robust glucose- or KCl-evoked fura-2-reported Ca^2+^ transients that were essentially abolished in cells expressing Ca_V_-aβlator (Figure 6e-g). Altogether, these results reveal the exceptional activity of Ca_V_-aβlator as a genetically-encoded HVACC inhibitor that is effective across diverse cellular contexts.

## Discussion

This work introduces Ca_V_-aβlator as a novel genetically-encoded molecule that potently inhibits HVACCs by targeting auxiliary Ca_V_β subunits. Ca_V_-aβlator combines the exquisite specificity of a Ca_V_β-targeted nanobody and the powerfully consequential catalytic activity of an E3 ubiquitin ligase. We discuss four distinct aspects of this work, based on viewing Ca_V_-aβlator from different perspectives; 1) as a unique tool to selectively erase HVACCs in cells, 2) as a method to probe mechanisms of HVACC regulation and trafficking, 3) as a potential therapeutic, and 4) as a prototype engineered protein that enables probing new dimensions of macro-molecular membrane protein signaling.

Ca^2+^ is a universal second messenger critical to the biology of virtually all cells. In excitable cells, both LVACCs and HVACCs transduce electrical signals encoded in action potentials into changes in intracellular Ca^2+^ that then drive many biological responses. In cells expressing both classes of channels, the physiological effects mediated specifically through LVACCs versus HVACCs in vivo can be difficult to decipher. Ca_V_-aβlator now presents as a tool that can be deployed in target cells to virtually erase all HVACCs while leaving LVACC actions intact. The closest existing proteins that can similarly eliminate HVACCs are RGK GTPases which are capable of potently inhibiting Ca_V_1/Ca_V_2 channels when over-expressed in target cells (Murata et al., 2004; Chen et al., 2005; Xu et al., 2010; Puckerin et al., 2018; Bannister et al., 2008). However, a distinct disadvantage of RGKs is their propensity for off-target effects due to their known interactions with, and regulation of, cytoskeletal proteins and other signaling molecules including 14-3-3, calmodulin, and CaM kinase II (Yang and Colecraft, 2013; Correll et al., 2008; Royer et al., 2018; Béguin et al., 2005). Over the last two decades, several groups have sought to disrupt the α_1_-Ca_V_β interaction with either small molecules or by over-expressing the AID peptide as a Ca_V_β sponge (Findeisen et al., 2017; Chen, 2018; Khanna et al., 2019; Yang et al., 2019). While this approach has shown some efficacy in certain instances, the potency of HVACC inhibition falls well short of that achieved here with Ca_V_-aβlator. Indeed, over-expressing the AID peptide in adult cardiac myocytes is not effective in inhibiting Ca_V_1.2 channels (Yang et al., 2019), because in this context α_1C_ binding to Ca_V_β is not absolutely required for channel trafficking to the surface (Yang et al., 2019; Meissner et al., 2011). Nevertheless, the ability of Ca_V_-aβlator to essentially eradicate *I*_Ca,L_ in adult cardiomyocytes indicates that under normal physiological conditions essentially all α_1C_ subunits are associated with a Ca_V_β in ventricular heart cells.

Ca_V_1/Ca_V_2 channels and other surface membrane proteins spend a significant portion of their life cycles in intracellular compartments reflecting their biogenesis, recycling, and ultimate destruction. The signals regulating HVACC degradation and trafficking among compartments are arcane and poorly understood, but likely prominently involve post-translational modifications of channel subunits. Here, we show that targeted ubiquitination of α_1C_/β_2_ complexes in cardiomyocytes with Ca_V_-aβlator specifically arrests Ca_V_1.2 channels in Rab7-positive late endosomes. Ca_V_-aβlator possesses the catalytic HECT domain of Nedd4L which is known to principally catalyze the addition of K63-linkage polyubiquitin chains to target proteins (Kim and Huibregtse, 2009; Scheffner and Kumar, 2014). Thus, our results suggest that K63-ubiquitin chains on α_1C_/β_2_ subunits may be a key signal directing Ca_V_1.2 channels to late endosomes. We further found that targeted ubiquitination of HVACC α_1_ subunits with Ca_V_-aβlator did not lead to their enhanced degradation either in heterologous cells or cardiomyocytes. By contrast, using a GFP nanobody to target the Nedd4L HECT domain to YFP-tagged KCNQ1, a known substrate of endogenous Nedd4L, resulted in reduced expression of this K^+^ channel pore-forming α_1_ subunit (Kanner et al., 2017). Hence, the impact of Nedd4L HECT domain on the stability of membrane proteins is likely substrate-dependent. We speculate that arming nb.F3 with the catalytic domains of other types of E3 ligases that catalyze formation of different polyubiquitin chains will elucidate the precise signals dictating Ca_V_1/Ca_V_2 channel degradation and trafficking among distinct compartments. Beyond ubiquitination, the approach could also be potentially used to elucidate functional consequences and mechanisms of other post-translational modifications such as phosphorylation/dephosphorylation on Ca_V_1/Ca_V_2 channels, as well as to localize sensors that report on signals within HVACC nano-domains in live cells.

Blocking the activity of specific HVACCs with small molecules is a prevailing or potential therapy for many cardiovascular and neurological diseases including; pain, hypertension, cardiac arrhythmias, epilepsy, and Parkinson’s disease (Zamponi, 2016). A limitation of small molecule or toxin blockers for HVACCs is the propensity for off-target effects due to their inevitable widespread distribution when administered to a patient. In some circumstances such off-target effects may limit the therapeutic window sufficiently to adversely affect treatment efficacy. Genetically-encoded HVACC inhibitors have great potential to be useful therapeutics with the advantage that their expression can be restricted to target tissues/cell types, or even to spatially discrete channels within single cells (Murata et al., 2004; Makarewich et al., 2012). Given its potency in silencing HVACC activity, Ca_V_-aβlator could be a lead molecule for future development into a gene therapy for particular applications where a genetically-encoded HVACC inhibitor is warranted. For this purpose, it may be desirable to generate Ca_V_-aβlator versions whose time course and extent of action could be tuned by either a small molecule or light. Indeed, this is a focus of ongoing work.

Finally, an exciting prospect is the potential of Ca_V_-aβlator as a prototype that can be further developed to engineer proteins that regulate Ca_V_1/Ca_V_2 channel complexes with new dimensions of specificity. For example, a prevailing idea is that Ca_V_1/Ca_V_2 channels of a particular type (e.g. Ca_V_1.2 channels in cardiomyocytes) may yet form discrete signaling units with different functional outputs in single cells based on their incorporation into divergent macro-molecular complexes (Shaw and Colecraft, 2013). There are tantalizing hints that different Ca_V_β isoforms could be a node of signal diversification by promoting formation of molecularly distinct HVACC macro-molecular complexes (McEnery et al., 1998; Brice and Dolphin, 1999; Campiglio and Flucher, 2015). Hence, the ability to inhibit specific Ca_V_ channel macro-molecular complexes based on the identity of the constituent Ca_V_β is biologically important, yet not rigorously addressable with conventional knockout/knockdown approaches. However, this capability may be readily achieved with Ca_V_-aβlators directed towards particular Ca_V_β isoforms. A challenge to realize this possibility is the development of Ca_V_β isoform-specific nanobodies which should be feasible given that there is sequence divergence among Ca_V_βs outside the conserved *src* homology 3 (SH3) and guanylate kinase (GK) domains (Buraei and Yang, 2010). In a broader context, the phenomenon of ion channel pore-forming α_1_ subunits assembled with diverse auxiliary subunits in individual cells is common throughout biology (O'Malley and Isom, 2015; Copits and Swanson, 2012; Trimmer, 2015). Hence, Ca_V_-aβlator-inspired molecules and approaches might be expected to elucidate functional dimensions of ion channel macro-molecular complex signaling that, to date, have remained refractory to analyses.

## Materials and methods

**Key resources table:** 

Reagent type (species) or resource	Designation	Source or reference	Identifiers	Additional information
Gene (rat)	CACNA1B		NM_147141	
Gene (rabbit)	CACNA1C		NM_001136522	
Gene (rat)	CACNB1		NM_017346	
Gene (human)	CACNB2		NM_201590	
Gene (rat)	CACNB3		NM_012828.2	
Gene (rat)	CACNB4		NM_001105733.1	
Gene (human)	CACNA2D1		NM_000722.4	
Gene (human)	NEDD4L		NM_001144965.2	
strain, strain background (*Escherichia coli*)	Rosetta DE3	Millipore Sigma		
Cell line (Human)	HEK293	Other	RRID: CVCL_0045	Laboratory of Dr. Robert Kass
Recombinant DNA reagent	nb.F3-CFP-PKC_γ_	This paper		Made by PCR, see molecular biology and cloning
Recombinant DNA reagent	nb.F3-P2A-CFP	This paper		Made by PCR, see molecular biology and cloning
Recombinant DNA reagent	nb.F3-Nedd4L-P2A-CFP	This paper		Made from pCI HA Nedd4L (Addgene #27000); see molecular biology and cloning
Recombinant DNA reagent	nb.F3-Nedd4L [C942S]-P2A-CFP	This paper		Made by site-directed mutagenesis
Recombinant DNA reagent	BBS-α_1B_	PMID: 20308247		
Recombinant DNA reagent	BBS-α_1C_	PMID: 20308247		
Antibody	Anti-α_1C_	Alomone	Cat#: ACC-003	1:1000 WB/IF
Antibody	Anti-α_1C_	NeuroMab	Clone: N263/31	1:200 IF
Antibody	Anti-Ca_V_β_1_	NeuroMab	Clone: N7/18	1:500 WB
Antibody	Anti-Ca_V_β_2_	Alomone	Cat#: ACC-105	1:200
Antibody	Anti-Rab5	Cell Signaling Technology	Cat#: 3547	1:200
Antibody	Anti-Rab7	Cell Signaling Technology	Cat#: 9367	1:200
Antibody	Anti-LAMP1	Developmental Studies Hybridoma Bank at the University of Iowa	RRID: AB_528127	1:100
Antibody	Anti-RyR	Thermo Fisher Scientific	Cat#: MA3-916	1:1000
Antibody	Anti-actin	Sigma	Cat#: A5060	1:1000
Antibody	Anti-ubiquitin, VU-1	LifeSensors	Cat#: VU101	1:500
Antibody	RFP-trap agarose beads	Chromotek	Cat#: rta-20	
Antibody	Anti-FLAG affinity gel	Sigma-Aldrich	Cat#: A2220	
Peptide, recombinant reagent	FLAG peptide	Sigma-Aldrich	Cat#: F3290	
Peptide, recombinant reagent	Ni-NTA agarose	Qiagen	Cat#: 30210	
Peptide, recombinant reagent	Protein A/G sepharose beads	Rockland		
Peptide, recombinant reagent	α-bungarotoxin, Alexa Fluor 647 conjugate	Life Technologies		
Peptide, recombinant reagent	Fura-2 AM	Life Technologies	Cat#: F1221	
chemical compound, drug	Phorbol 12,13-dibutyrate	Sigma-Aldrich	Cat#: P1269	
commercial assay or kit	AdEasy Adenoviral Vector Systems	Stratagene		
commercial assay or kit	QuikChange Lightning Site-Directed Mutagenesis Kit	Stratagene		
software, algorithm	FlowJo		RRID: SCR_008520	
software, algorithm	PulseFit	HEKA		
software, algorithm	EasyRatioPro	HORIBA		
software, algorithm	GraphPad Prism		RRID: SCR_002798	

### Protein purification

We used the BacMam expression system to purify Ca_V_β_1B_ and Ca_V_β_3_ (Goehring et al., 2014). Briefly, full-length Ca_V_β_1b_ and Ca_V_β_3_ were cloned into a modified pEG BacMam vector with a C-terminal FLAG tag using BamHI and EcoRI sites. BacMam virus was subsequently generated in Sf9 cells and harvested after three rounds of amplification. 100 mL of BacMam virus was used to infect 1 L of HEK293 GnTI^-^ cells (N-acetylglucosaminyltransferase I-negative) and kept shaking at 37°C. After 18 hrs the cells were stimulated with 10 mM sodium butyrate and harvested 72 hrs later. Cells were lysed using an Avestin Emulsiflex-C3 homogenizer in buffer containing 50 mM Tris, 150 mM KCl, 10% sucrose, 1 mM PMSF (phenylmethylsulfonyl fluoride), and EDTA-free Complete protease inhibitor cocktail (Roche), pH 7.4. Lysate was spun down at 35,000 g for 1 hr. Ca_V_β was subsequently isolated from supernatant with anti-FLAG antibody (M2) affinity chromatography, and eluted with 100 µg/mL FLAG peptide (Sigma Millipore) in 50 mM TrisHCl, 150 mM KCl, pH 7.4. The protein was then applied to an ion exchange column (MonoQ, GE) and eluted with a linear KCl gradient of 50 mM to 1M. Peak fractions were collected and subjected to size exclusion chromatography (Superdex 200, GE) in a buffer containing 20 mM Tris, 150 mM KCl, pH 7.4. Proteins were brought to 20% glycerol, flash frozen, and stored at −80°C.

For isothermal titration calorimetry experiments, both Ca_V_β_2b_ and nb.F3 were cloned via Gibson assembly (Gibson et al., 2009) into an IPTG (isopropyl β-D-1-thiogalactopyranoside) inducible, kanamycin-resistant pET derived plasmid (Novagen, Madison, Wisconsin), with an N-terminal deca-histidine tag (His10) and transformed into Rosetta DE3 *E. coli* (Millipore Sigma), following manufacturers’ instructions. Cells were grown at 37°C in 1L 2xTY media supplemented with 50 ug/mL carbenicillin and 35 μg/mL chloramphenicol and shook at 225 rpm. Protein expression was induced with 0.2 mM IPTG when the cells reached an OD of 0.6–0.8. The cells were then grown overnight at 22°C.

Nb.F3 was purified as previously described (McMahon et al., 2018): briefly, cells were harvested and resuspended in 100 mL buffer containing (mM) 500 sucrose, 200 Tris (pH 8), 0.5 EDTA and osmotically shocked with the addition of 200 mL water with stirring. The lysate was brought to a concentration of (mM) 150 NaCl, 2 MgCl_2_, and 20 imidazole and centrifuged at 20,000 g, 4°C for 30 min. The supernatant was combined with 2 mL Ni-NTA Sepharose resin (Qiagen) in batch, washed with 70 mM imidazole, and eluted with 350 mM imidazole. The eluant was dialyzed into a buffer containing 150 mM NaCl, 10 mM HEPES, pH 7.4 and purified with an S200 size exclusion column (GE Healthcare).

For the purification of Ca_V_β_2b_, cells were pelleted and resuspended in a buffer containing (mM) 300 NaCl, 20 Tris HCl, 10% glycerol, pH 7.4, 0.5 PMSF, and EDTA-free Complete protease inhibitor cocktail (Roche). Cells were lysed using an Avestin Emulisflex-C3 homogenizer and spun at 35,000 g for 30’. The solubilized protein was applied to Ni-NTA Sepharose (Qiagen) and purified as nb.F3.

### Nanobody generation

One llama was immunized with an initial injection of 600 µg purified Ca_V_β_1b_ and Ca_V_β_3_, with four boosters of 200 ug each protein administered every other week (Capralogics Inc, Hardwick, MA). 87 days after the first immunization, lymphocytes were isolated from blood and a cDNA library with ProtoScript II Reverse Transcriptase (New England Biolabs). Nanobodies were isolated as previously described (Pardon et al., 2014), using a two-step nested PCR. Amplified Vhh genes were cloned into the phagemid plasmid pComb3xSS, a gift from Carlos Barbas (Andris-Widhopf et al., 2000) (Addgene plasmid # 63890). A phage display library was created using electrocompetent TG1 *E. coli* cells (Lucigen). Three rounds of phage display were performed as previously described (Pardon et al., 2014), using 100 nM biotinylated Ca_V_β_3_ as bait on neutravidin-coated Nunc-Immuno plates (Thermo Scientific). Clones of interest were subsequently cloned into mammalian expression systems for further study (see below).

### Isothermal titration calorimetry

Isothermal Titraction calorimetry measurements were performed using an MicroCal Auto iTC 200 (Malvern Panalytical) at 25°C. Samples were dialyzed into 300 mM NaCl, 20 mM HEPES, 5% glycerol, pH 7.5 and filtered beforehand. Injections of 2 µL nb.F3 into 400 µL of Ca_V_β_2b_. Data were processed with MicroCal Origin 7.0.

### Molecular biology and plasmid construction

Potential nbs were PCR amplified with primers flanking their conserved framework (FW) FW1 and FW4 regions and inserted into the mammalian expression plasmid pcDNA3 (Invitrogen) using HindIII and EcoRI sites. An additional GSG linker was included in the PCR and the insert was ligated upstream of an enhanced CFP and C1 domain of human PKCγ (residues 51–180).

Rat Ca_V_β_1b_, a kind gift from Dr. Jian Yang (Columbia University), was PCR amplified for subsequent overlap PCR with YFP, inserting a GSG linker between the two proteins. The resulting Ca_V_β_1b_-GSG-YFP sequence was digested with BamHI and NotI and ligated into a PiggyBac CMV mammalian expression vector (System Biosciences). A similar cloning strategy was used for Ca_V_β_3_ and Ca_V_β_4_. Rat Ca_V_β_2a_ was PCR amplified with an N-terminal YFP to prevent palmitoylation of the β_2a_ subunit (Chien et al., 1996) and inserted with a similar strategy.

A customized bicistronic vector (xx-P2A-CFP) was synthesized in the pUC57 vector, in which coding sequence for P2A peptide was sandwiched between an upstream multiple cloning site and enhanced cyan fluorescent protein (CFP) (Genewiz). The xx-P2A-CFP fragment was amplified by PCR and cloned into the PiggyBac CMV mammalian expression vector (System Biosciences) using NheI/NotI sites. To generate nb.F3 -P2A-CFP, we PCR amplified the coding sequence for nb.F3 and cloned it into xx-P2A-CFP using NheI/AflII sites. A similar backbone was created in the PiggyBac CMV mammalian expression vector in which CFP-P2A-xx contained a multiple cloning site downstream of the P2A site (Genewiz). Nb.F3 was PCR amplified and ligated into the vector with BglII/AscI sites. The HECT domain of human Nedd4L (Gao et al., 2009) (a gift from Joan Massague, Addgene plasmid # 27000) consisting of residues 594–974 was PCR amplified and inserted downstream of nb.F3 using AscI/AgeI sites. Mutagenesis of C942S was accomplished using site-directed mutagenesis.

α_1B_-BBS, harboring two tandem 13 residue bungarotoxin-binding sites (SWRYYESSLEPYPD) in the domain IV S5-S6 extracellular loop, was a kind gift from Dr. Steven Ikeda (NIAAA). α_1C_ and α_1C_-BBS, and α_1C_-BBS-YFP have been described previously (Yang et al., 2010; Kanner et al., 2017).

### Cell culture and transfection

Human embryonic kidney (HEK293) cells were a kind gift from the laboratory of Dr. Robert Kass (Columbia University). Cells were mycoplasma free, as determined by the MycoFluor Mycoplasma Detection Kit (Invitrogen, Carlsbad, CA). Low passage HEK293 cells were cultured at 37°C in DMEM supplemented with 5% fetal bovine serum (FBS) and 100 mg/mL of penicillin–streptomycin. HEK293 cell transfection was accomplished using the calcium phosphate precipitation method. Briefly, plasmid DNA was mixed with 7.75 μL of 2 M CaCl_2_ and sterile deionized water (to a final volume of 62 μL). The mixture was added dropwise, with constant tapping to 62 μL of 2x Hepes buffered saline containing (in mM): Hepes 50, NaCl 280, Na_2_HPO_4_ 1.5, pH 7.09. The resulting DNA–calcium phosphate mixture was incubated for 20 min at room temperature and then added dropwise to HEK293 cells (60–80% confluent). Cells were washed with Ca^2+^-free phosphate buffered saline after 4–6 hr and maintained in supplemented DMEM.

Isolation of adult guinea pig cardiomyocytes was performed in accordance with the guidelines of Columbia University Animal Care and Use Committee. Prior to isolation, plating dishes were precoated with 15 µg/mL laminin (Gibco). Adult female Hartley guinea pigs (Charles River) were euthanized with 5% isoflurane, hearts were excised and ventricular myocytes isolated by first perfusing in KH solution (mM): 118 NaCl, 4.8 KCl, 1 CaCl_2_ 25 HEPES, 1.25 K_2_HPO_4_, 1.25 MgSO_4_, 11 glucose,. 02 EGTA, pH 7.4, followed by KH solution without calcium using a Langendorff perfusion apparatus. Enzymatic digestion with 0.3 mg/mL Collagenase Type 4 (Worthington) with 0.08 mg/mL protease and. 05% BSA was performed in KH buffer without calcium for six minutes. After digestion, 40 mL of a high K^+^ solution was perfused through the heart (mM): 120 potassium glutamate, 25 KCl, 10 HEPES, 1 MgCl_2_, and. 02 EGTA, pH 7.4. Cells were subsequently dispersed in high K^+^ solution. Healthy rod-shaped myocytes were cultured in Medium 199 (Life Technologies) supplemented with (mM): 10 HEPES (Gibco), 1x MEM non-essential amino acids (Gibco), 2 L-glutamine (Gibco), 20 D-glucose (Sigma Aldrich), 1% vol vol^−1^ penicillin-streptomycin-glutamine (Fisher Scientific),. 02 mg/mL Vitamin B-12 (Sigma Aldrich) and 5% (vol/vol) FBS (Life Technologies) to promote attachment to dishes. After 5 hr, the culture medium was switched to Medium 199 with 1% (vol/vol) serum, but otherwise supplemented as described above. Cultures were maintained in humidified incubators at 37°C and 5% CO_2_.

Murine dorsal root ganglion (DRG) neurons were kindly provided by the laboratory of Dr. Ellen Lumpkin (Columbia University). DRG neurons were isolated as previously described (Albuquerque et al., 2009). DRG neurons were plated onto glass coverslips coated with 15 µg/mL laminin (Corning) and maintained in Neurobasal media (Thermo Fisher Scientific) supplemented with 1x B-27 (Thermo Fisher Scientific), 100 µg mL^−1^ penicillin/streptomycin (Fisher Scientific), 0.29 mg/mL L-glutamine (Gibco), 50 ng mL^−1^ NGF (Sigma Aldrich), 2 ng mL^−1^ GDNF (Sigma Aldrich), and 10 µM cytosine β-D-arabinofuranoside (Sigma Aldrich).

### Pancreatic beta cell isolation and culture

Murine pancreatic β-cells from Rip-Cre (Jackson Laboratories Stock #003573) mice crossed with Rosa26-tdTomato (Jackson Laboratories Stock #007909) mice were kindly provided by the laboratory of Dr. Domenico Accili (Columbia University). Islets were isolated as previously described (Stull et al., 2012), dispersed with 0.05% trypsin EDTA (Gibco) and plated onto 35 mm glass bottom dishes with 10 mm microwells (Cellvis) pre-coated with 10 mg/mL fibronectin (Sigma Aldrich). Islets were maintained in RPMI 1640 media (Corning) supplemented with 15% FBS and 100 µg mL^−1^ penicillin/streptomycin. Islets were imaged 24–48 hr after adenoviral infection.

### Adenoviral generation

Adenoviral vectors expressing GFP and CFP-P2A-nb.F3-Nedd4L[C942S] were generated using the pAdEasy system (Stratagene) according to manufacturer’s instructions as previously described (Kanner et al., 2017; Subramanyam, 2013). Plasmid shuttle vectors (pShuttle CMV) containing cDNA for CFP-P2A-nb.F3-Nedd4L[C942S] were linearized with PmeI and electroporated into BJ5183-AD-1 electrocompetent cells pre-transformed with the pAdEasy-1 viral plasmid (Stratagene). PacI restriction digestion was used to identify transformants with successful recombination. Positive recombinants were amplified using XL-10-Gold bacteria, and the recombinant adenoviral plasmid DNA linearized with PacI digestion. HEK cells cultured in 60 mm diameter dishes at 70–80% confluency were transfected with PacI-digested linearized adenoviral DNA. Transfected plates were monitored for cytopathic effects (CPEs) and adenoviral plaques. Cells were harvested and subjected to three consecutive freeze-thaw cycles, followed by centrifugation (2,500 × g) to remove cellular debris. The supernatant (2 mL) was used to infect a 10 cm dish of 90% confluent HEK293 cells. Following observation of CPEs after 2–3 d, cell supernatants were used to re-infect a new plate of HEK293 cells. Viral expansion and purification was carried out as previously described (Colecraft et al., 2002). Briefly, confluent HEK293 cells grown on 15 cm culture dishes (x8) were infected with viral supernatant (1 mL) obtained as described above. After 48 hr, cells from all of the plates were harvested, pelleted by centrifugation, and resuspended in 8 mL of buffer containing (in mM) 20 Tris HCl, 1 CaCl_2_, one and MgCl_2_ (pH 8). Cells were lysed by four consecutive freeze-thaw cycles and cellular debris pelleted by centrifugation. The virus-laden supernatant was purified on a cesium chloride (CsCl) discontinuous gradient by layering three densities of CsCl (1.25, 1.33, and 1.45 g/mL). After centrifugation (50,000 rpm; SW41Ti Rotor, Beckman-Coulter Optima L-100K ultracentrifuge; 1 hr, 4°C), a band of virus at the interface between the 1.33 and 1.45 g/mL layers was removed and dialyzed against PBS (12 hr, 4°C). Adenoviral vector aliquots were frozen in 10% glycerol at −80°C until use. Generation of CFP-P2A-nb.F3-Nedd4L was performed by Vector Biolabs (Malvern, PA).

### Flow cytometry assay of total and surface calcium channels

Cell surface and total ion channel pools were assayed by flow cytometry in live, transfected HEK293 cells as previously described (Kanner et al., 2017; Aromolaran et al., 2014). Briefly, 48 hr post-transfection, cells cultured in 12-well plates were gently washed with ice cold PBS containing Ca^2+^ and Mg^2+^ (in mM: 0.9 CaCl_2_, 0.49 MgCl_2_, pH 7.4), and then incubated for 30 min in blocking medium (DMEM with 3% BSA) at 4°C. HEK293 cells were then incubated with 1 μM Alexa Fluor 647 conjugated α-bungarotoxin (BTX_647_; Life Technologies) in DMEM/3% BSA on a rocker at 4°C for 1 hr, followed by washing three times with PBS (containing Ca^2+^ and Mg^2+^). Cells were gently harvested in Ca^2+^-free PBS, and assayed by flow cytometry using a BD Fortessa Cell Analyzer (BD Biosciences, San Jose, CA, USA). CFP- and YFP-tagged proteins were excited at 407 and 488 nm, respectively, and Alexa Fluor 647 was excited at 633 nm.

### Electrophysiology

Whole-cell recordings of HEK293 cells were conducted 48 hr after transfection using an EPC-10 patch clamp amplifier (HEKA Electronics) controlled by Pulse software (HEKA). Micropipettes were prepared from 1.5 mm thin-walled glass (World Precision Instruments) using a P97 microelectrode puller (Sutter Instruments). Internal solution contained (mM): 135 cesium-methansulfonate (CsMeSO_3_), 5 CsCl, 5 EGTA, 1 MgCl_2_, 2 MgATP, and 10 HEPES (pH 7.3). Series resistance was typically between 1–2 MΩ. There was no electronic resistance compensation. External solution contained (mM): 140 tetraethylammonium-MeSO_3_, 5 BaCl_2_, and 10 HEPES (pH 7.4). Whole-cell I-V curves were generated from a family of step depolarizations (−60 mV to +80 mV from a holding potential of −90 mV). Currents were sampled at 20 kHz and filtered at 5 kHz. Traces were acquired at a repetition interval of 10 s. Leak and capacitive transients were subtracted using a P/4 protocol.

Whole-cell recordings of cardiomyocytes and DRG neurons were performed 48 hr after infection. HEK cell internal and external solutions were used for DRG experiments. Whole-cell recordings for guinea pig cardiomyocytes used internal solution comprised of (mM): 150 CsMeSO_3_, 10 EGTA, 5 CsCl, MgCl_2_, 4 MgATP, and 10 HEPES. For formation of gigaohm seals and initial break-in to the whole-cell configuration, cells were perfused in Tyrode solution containing (mM): 138 NaCl, 4 KCl, 2 CaCl_2_, 1 MgCl_2_, 0.33 NaH_2_PO_4_, and 10 HEPES (pH 7.4). Upon successful break-in, the perfusing media was switched to an external solution composed of (mM): 155 *N*-methyl-D-glucamine, 10 4-amino-pyridine, 1 MgCl_2_, 5 BaCl_2_, and 10 HEPES (pH 7.4). Currents were sampled at 20 kHz and filtered at 5 kHz. Leak and capacitive transients were subtracted using a P/4 protocol.

### Immunofluorescence staining

Approximately 48 hr after adenoviral infection, guinea pig cardiomyocytes were fixed in 4% paraformaldehyde (wt/vol, in PBS) for 20 min at RT. Cells were washed twice with PBS and then incubated in 0.1M glycine (in PBS) for 10 min at RT to block free aldehyde groups. Fixed cells were then permeabilized with 0.2% Triton X-100 (in PBS) for 20 min at RT. Non-specific binding was blocked with a 1 hr incubation at RT in PBS solution containing 3% (vol vol^−1^) normal goat serum (NGS), 1% BSA, and 0.1% Triton X-100. Cells were then incubated with primary antibody in PBS containing 1% NGS, 1% BSA, and 0.1% BSA overnight at 4°C. Cells were washed three times for 10 min each with PBS with 0.1% Triton X-100 and then stained with secondary antibody for 1 hr at RT. Antibody dilutions were prepared in PBS solution containing 1% NGS, 1% BSA, and 0.1% Triton X-100. The cells were then washed in PBS with 0.1% Triton X-100 and imaged in the same solution. Primary antibodies and working dilutions were as follows: α_1C_: Alomone, 1:1000; UC Davis/NIH NeuroMab Facility, clone N263/31, 1:200. RyR: Sigma Aldrich, 1:1000. Ca_V_β_2_: Alomone, 1:200. Rab7: Cell Signaling Technology, 1:100. Rab5: Cell Signaling Technology, 1:200. Lamp1: Developmental Studies Hybridoma Bank, created by the NICHD of the NIH and maintained at The University of Iowa, Department of Biology, Iowa City, IA 52242, 1:100. Secondary antibodies (Thermofisher) were used at a dilution of 1:1000.

### Confocal microscopy

Cells were plated onto 35 mm MatTek imaging dishes (MatTek Corporation). Images were captured on a Nikon A1RMP confocal microscope with a 40x oil immersion objective (1.3 N.A.). CFP, Alexa-488, YFP, and Alexa-647 were imaged using 458, 488, 514 and 639 nm laser lines, respectively.

### Pulldown assays

Transfected HEK293 cells cultured in 60 mm dishes were harvested in PBS, centrifuged at 2,000 g (4°C) for 5 min, and the pellet resuspended in RIPA lysis buffer containing (mM): 150 NaCl, 20 Tris HCl, 1 EDTA, 0.1% (wt vol^−1^) SDS, 1% Triton X-100, 1% sodium deoxycholate, and supplemented with protease inhibitor mixture (10 µL mL^−1^, Sigma Aldrich), 1 PMSF, 2 N-ethylmaleimide,. 05 PR-619 deubiquitinase inhibitor (LifeSensors). Cells were lysed on ice for 1 hr with intermittent vortexing and centrifuged at 10,000 g for 10 min (4°C). The soluble lysate collected and protein concentration determined with the bis-cinchonic acid protein estimation kit (Pierce Technologies).

For Ca_V_β_1b_ pulldowns, lysates were precleared with 10 µL of protein A/G sepharose beads (Rockland) for 1 hr at 4°C and then incubated with 2 µg anti-Ca_V_β_1_ antibody (UC Davis/NIH NeuroMab Facility, clone N7/18) for 1 hr at 4°C. Equivalent amounts of protein were then added to spin columns with 25 μL equilibrated protein A/G sepharose beads and rotated overight at 4°C. Immunoprecipitates were washed a total of five times with RIPA buffer and then eluted with 30 μL elution buffer (50 mM Tris, 10% (vol vol^−1^) glycerol, 2% SDS, 100 mM DTT, and 0.2 mg mL^−1^ bromophenol blue) at 55°C for 15 min. For α_1C_ pulldowns, lysates were added to spin columns containing 10 μL of equilibrated RFP-trap agarose beads, rotated at 4°C for 1 hr, and then washed/eluted as described above. Proteins were resolved on a 4–12% Bis Tris gradient precast gel (Life Technologies) in MOPS-SDS running buffer (Life Technologies) at 200 V constant for ~1 hr. Protein bands were transferred by tank transfer onto a polyvinylidene difluoride (PVDF, EMD Millipore) membrane in transfer buffer (25 mM Tris pH 8.3, 192 mM glycine, 15% (vol/vol) methanol, and 0.1% SDS). The membranes were blocked with a solution of 5% nonfat milk (BioRad) in Tris-buffered saline-tween (TBS-T) (25 mM Tris pH 7.4, 150 mM NaCl, and 0.1% Tween-20) for 1 hr at RT and then incubated overnight at 4°C with primary antibodies (Ca_V_β_1_, UC Davis/NIH NeuroMab Facility. Actin, Sigma Aldrich) in blocking solution. The blots were washed with TBS-T three times for 10 min each and then incubated with secondary horseradish peroxidase-conjugated antibody for 1 hr at RT. After washing in TBS-T, the blots were developed with a chemiluminiscent detection kit (Pierce Technologies) and then visualized on a gel imager. Membranes were then stripped with harsh stripping buffer (2% SDS, 62 mM Tris pH 6.8, 0.8% ß-mercaptoethanol) at 50°C for 30 min, rinsed under running water for 2 min, and washed with TBST (3x, 10 min). Membranes were pre-treated with 0.5% glutaraldehyde and re-blotted with anti-ubiquitin (VU1, LifeSensors) as per the manufacturers’ instructions.

### Calcium imaging

DRG neurons were washed twice in basal solution containing (mM): 145 NaCl, 5 KCl, 2 CaCl_2_, 1 MgCl_2_, one sodium citrate, 10 HEPES, 10 D-glucose, pH 7.4, and incubated in the same solution containing 5 uM fura-2 with 0.05% Pluronic F-127 detergent (Life Technologies) for 1 hr at 37°C, 5% CO_2_. Afterwards, cells were washed twice in same solution and placed on an inverted Nikon Ti-eclipse microscope with a Nikon Plan fluor 20x objective (0.45 N.A.). Fura-2 measurements were recorded at excitation wavelengths of 340 and 380 nm using EasyRatioPro (HORIBA Scientific). DRG neurons were depolarized with a solution in which NaCl was reduced to 110 mM and KCl increased to 40 mM.

Pancreatic β-cells were imaged with a similar protocol. Cells were maintained in a basal KRBH solution composed of (mM): 134 NaCl, 3.5 KCl, 1.2 KH_2_PO_4_, 0.5 MgSO_4_, 1.5 CaCl_2_, 5 NaHCO3, 10 HEPES, 2.8 D-glucose, pH 7.4. Stimulation solutions included either 16.8 mM glucose or 40 mM KCl, with NaCl concentrations adjusted accordingly to balance osmolarity with KRBH solution.

### Data and statistical analysis

Data were analyzed off-line using FloJo, PulseFit (HEKA), Microsoft Excel, Origin and GraphPad Prism software. Statistical analyses were performed in Origin or GraphPad Prism using built-in functions. Statistically significant differences between means (p<0.05) were determined using Student’s *t* test for comparisons between two groups or one-way ANOVA for three groups, with Tukey’s post-hoc analysis. Data are presented as means ± s.e.m.

## Data Availability

All data generated or analysed during this study are included in the manuscript and supporting files.

## Additional files

Transparent reporting form
